# Supplementary material for: Nicotine-mediated OTUD3 downregulation inhibits VEGF-C mRNA decay to promote lymphatic metastasis of human esophageal cancer
Source: Nat Commun. 2021 Dec 1;12:7006. doi: 10.1038/s41467-021-27348-8 (PMC8636640; doi:10.1038/s41467-021-27348-8)
Supplement: Supplementary file 1 — Supplementary Information [file 41467_2021_27348_MOESM1_ESM.pdf]

Supplementary Information

Nicotine-mediated OTUD3 downregulation inhibits VEGF-C mRNA decay to promote lymphatic metastasis of human esophageal cancer

Wang et al.

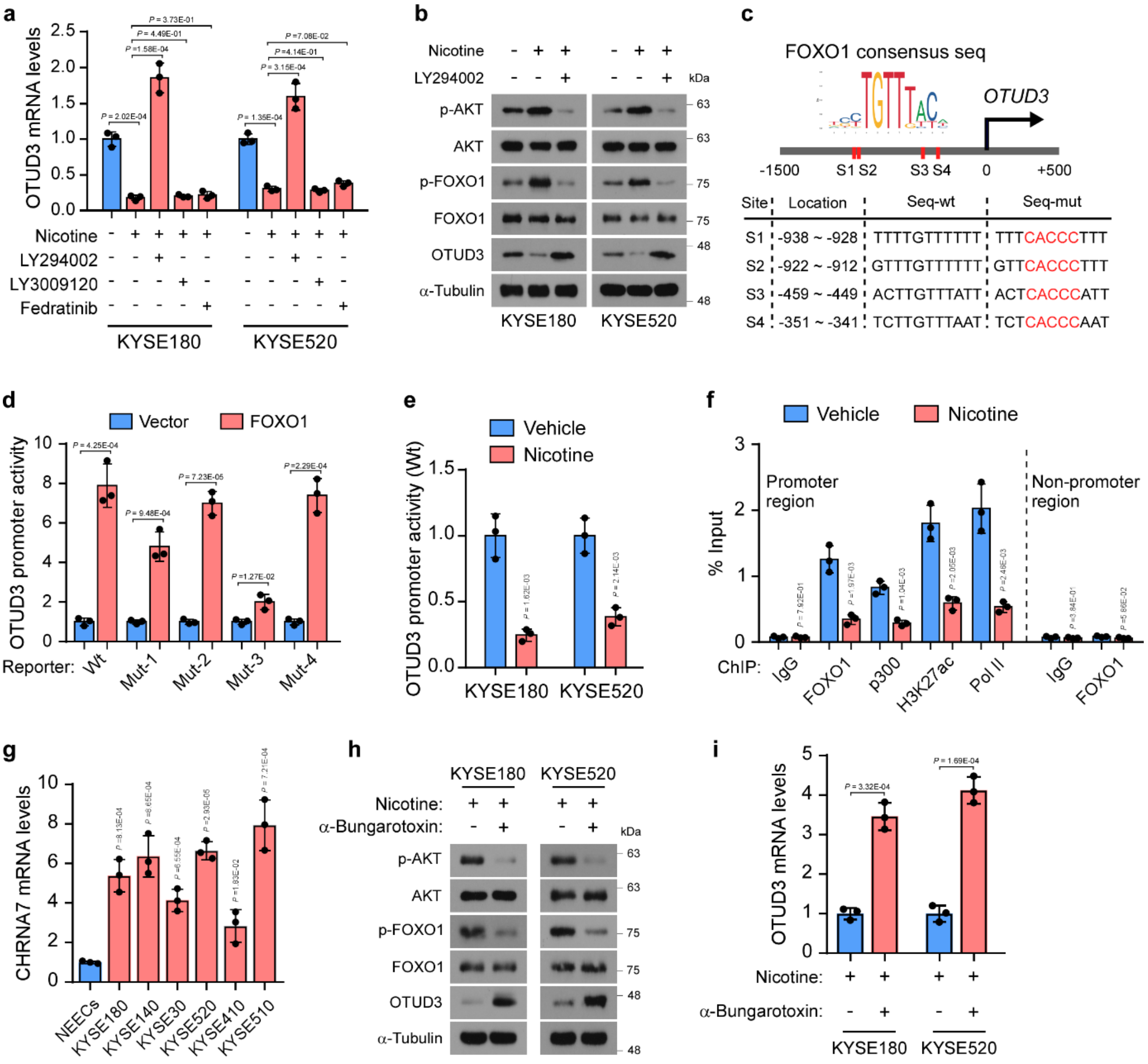

Supplementary Fig. 1. a qRT-PCR analysis of OTUD3 mRNA in KYSE180 and KYSE520 cells treated with nicotine (2 µM) alone, or co-administration with PI3K inhibitor LY294002 (500nM), or RAF inhibitor LY3009120 (20nM), or JAK2 inhibitor Fedratinib (3nM). **b** Western blot analysis of indicated proteins in esophageal cancer cells under treatment of nicotine or co-administration with LY294002. Immunoblot is representative of three biological replicates. **c** The putative *OTUD3* promoter (-1500 to +500 bp) contains four potential FOXO1-binding sites. The schematic illustration showed predicted binding sites of FOXO1. Mutations of binding sites in the *OTUD3* promoter were indicated. **d** Luciferase assays examined the activity of wild-type (wt) or site-mutant *OTUD3* promoter reporters in Vector and FOXO1-overexpressing KYSE180 cells. **e** Relative luciferase activities of *OTUD3* promoter in KYSE180 cells treated with vehicle or nicotine. **f** The enrichment of FOXO1, p300 acetyltransferase, H3K27ac, and RNA polymerase II (Pol II) on the *OTUD3* promoter in KYSE180 cells treated with vehicle or nicotine was determined by ChIP assays. The non-promoter region "-6297~-6202" was used as a negative control. **g** qRT-PCR analysis of CHRNA7 mRNA in normal esophageal epithelial cell (NEEC) and six esophageal cancer cell lines. **h** Western blot analysis of indicated proteins in the nicotine-treated KYSE180 and KYSE520 cells with or without co-administration of the alpha7 nAChR antagonist α-Bungarotoxin (5 µg/ml). Immunoblot is representative of three biological replicates. **i** qRT-PCR analysis of OTUD3 mRNA in the nicotine-treated KYSE180 and KYSE520 cells with or without co-administration of the alpha7 nAChR antagonist α-Bungarotoxin (5 µg/ml). Each error bar in panels **a**, **d-g** and **i** represents the mean±SD of three biological replicates. Two-sided Student's t-test was used for statistical analysis. Source data are provided as a Source Data file.

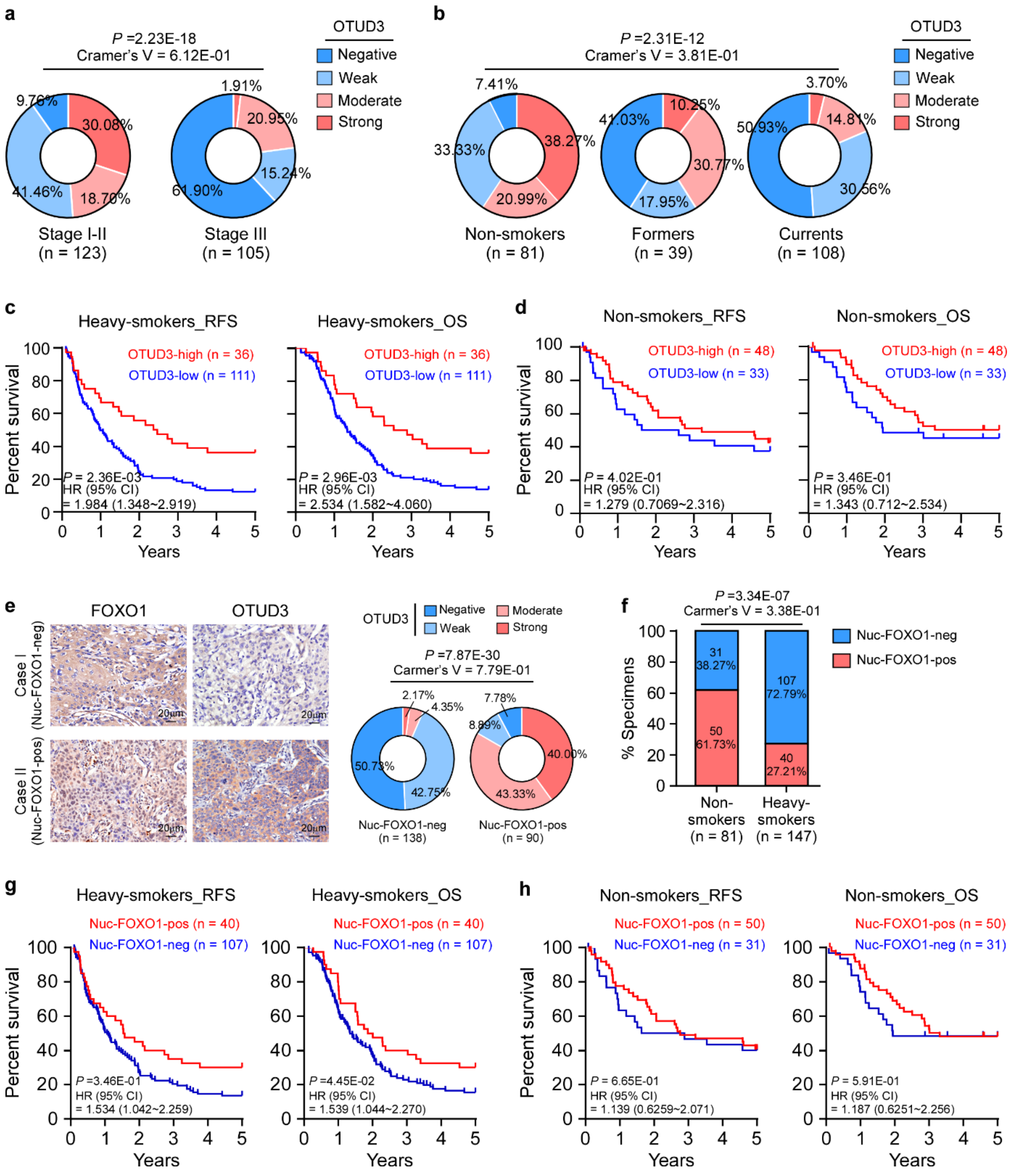

Supplementary Fig. 2. a The distribution of OTUD3 staining between clinical stage I-II (123) and stage III (105) was compared. Two-sided χ^2 test and Cramer's V were used to evaluate the correlation. **b** The correlation between OTUD3 expression scores and smoking status (non-smokers (n = 81), former smokers (n = 39), and current smokers (n = 108)) of esophageal cancer patients was compared. Two-sided χ^2 test and Cramer's V were used to evaluate the correlation. **c-d** RFS and OS analyses in heavy-smoking (c) or non-smoking (d) esophageal cancer patients stratified by low and high OTUD3 expression. Log-rank test was used for statistical analysis. **e** Representative images of FOXO1 and OTUD3 IHC staining in esophageal cancer specimens (n = 228). The correlation between nuclear FOXO1 expression and OTUD3 expression was tested using two-sided χ^2 test. **f** The correlation between smoking behaviors and nuclear FOXO1 expression was analyzed (two-sided χ^2 test). **g-h** RFS and OS analyses in heavy-smoking (g) or non-smoking (h) esophageal cancer patients stratified by negative or positive nuclear FOXO1 expression. Log-rank test was used for statistical analysis. Source data are provided as a Source Data file.

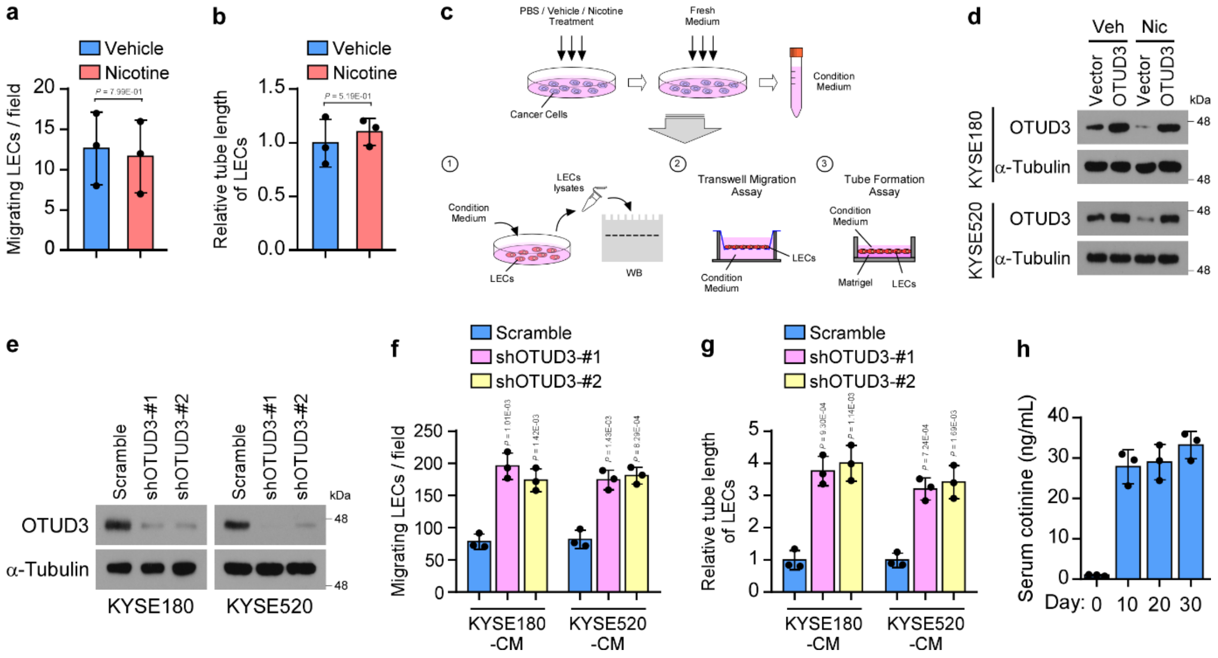

Supplementary Fig. 3. a Quantification of migrating human lymphatic endothelial cells (LECs) treated with vehicle or nicotine. **b** Relative tube length of LECs stimulated with vehicle or nicotine. **c** Scheme of conditioned-medium (CM) treatment experiments for analysis of tumor-induced lymphangiogenesis. **d** Western blot analysis of OTUD3 in Vector or OTUD3-overexpressing KYSE180 and KYSE520 cells with vehicle or nicotine treatment. **e** Western blot analysis of OTUD3 in scramble or OTUD3-silencing KYSE180 and KYSE520 cells. **f** Quantification of migrating LECs treated with indicated CM. **g** Relative tube length of LECs under treatment of indicated CM. **h** ELISA assays examined cotinine concentration in mice serum at days 0, 10, 20 and 30. Each error bar in panels **a**, **b**, and **f-h** represents the mean±SD of three biological replicates. Two-sided Student's t-test was used for statistical analysis. Source data are provided as a Source Data file.

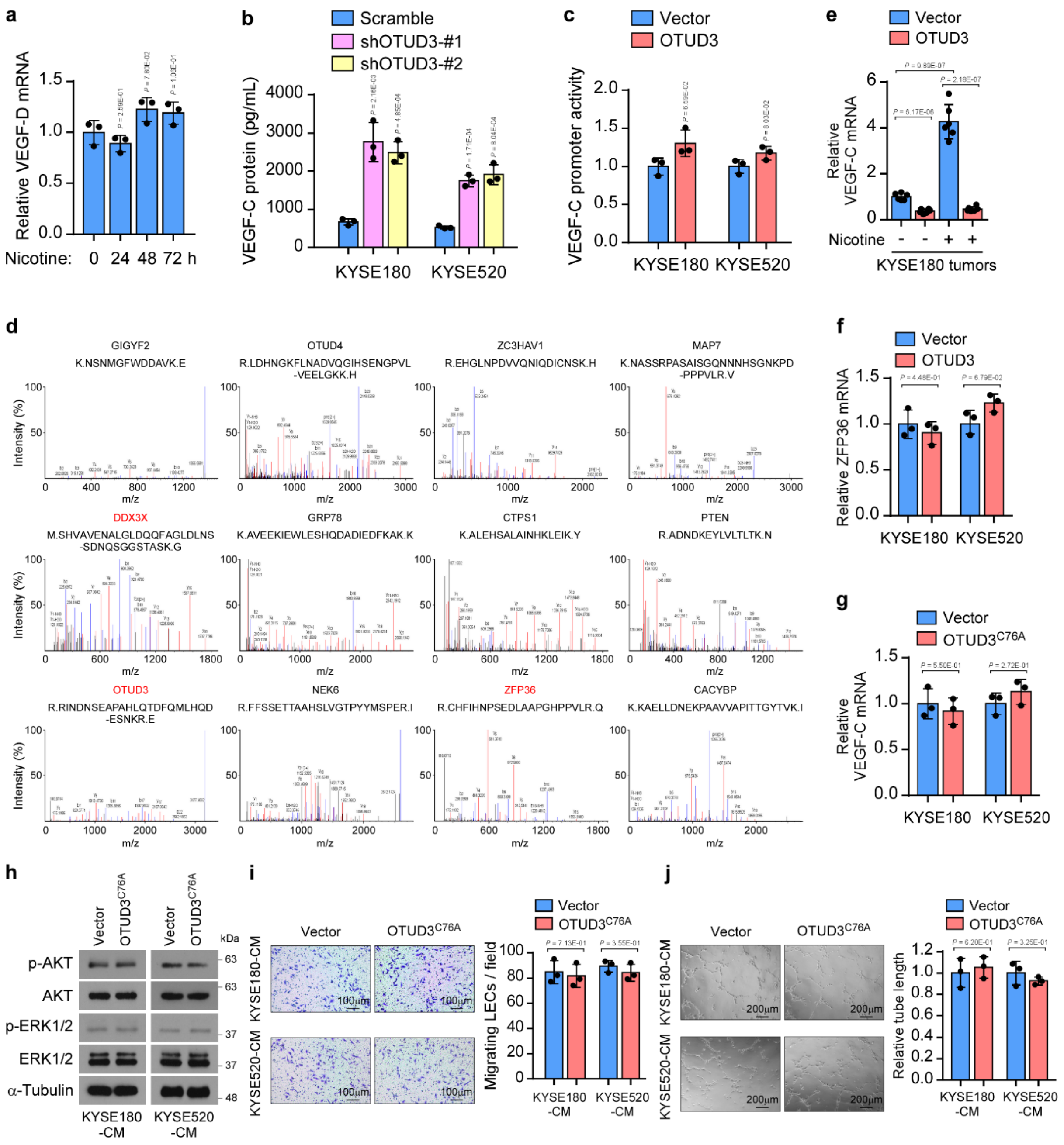

Supplementary Fig. 4. a KYSE180 cells were treated with vehicle or nicotine for the indicated time and then subjected to qRT-PCR analysis of VEGF-D. **b** ELISA assays examined the secreted VEGF-C concentration in the CM derived from scramble or OTUD3-silencing esophageal cancer cells. **c** Luciferase activities of *VEGF-C* promoter in Vector or OTUD3-overexpressing KYSE180 and KYSE520 cells. **d** Representative MS plots of OTUD3-interacting proteins were indicated. The mass spectrometry data have been deposited in the iProX database (#PXD028751). **e** qRT-PCR analysis of VEGF-C in indicated footpad tumors (n = 6, two-sided Student's t-test). **f** qRT-PCR analysis of ZFP36 in KYSE180 and KYSE520 cells with or without OTUD3 overexpression. **g** qRT-PCR analysis of VEGF-C in Vector or OTUD3^{C76A} transfected KYSE180 and KYSE520 cells. **h** LECs were treated with CM from Vector or OTUD3^{C76A} KYSE180 and KYSE520 cells and then subjected to western blot analysis of indicated proteins. **i** Representative images and quantification of migrating LECs treated with indicated CM. **j** Representative images and quantification of tube formation of LECs under treatment of indicated CM. Each error bar in panels **a-c**, **e-g**, **i,** and **j** represents the mean±SD of three biological replicates. Two-sided Student's t-test was used for statistical analysis. Source data are provided as a Source Data file.

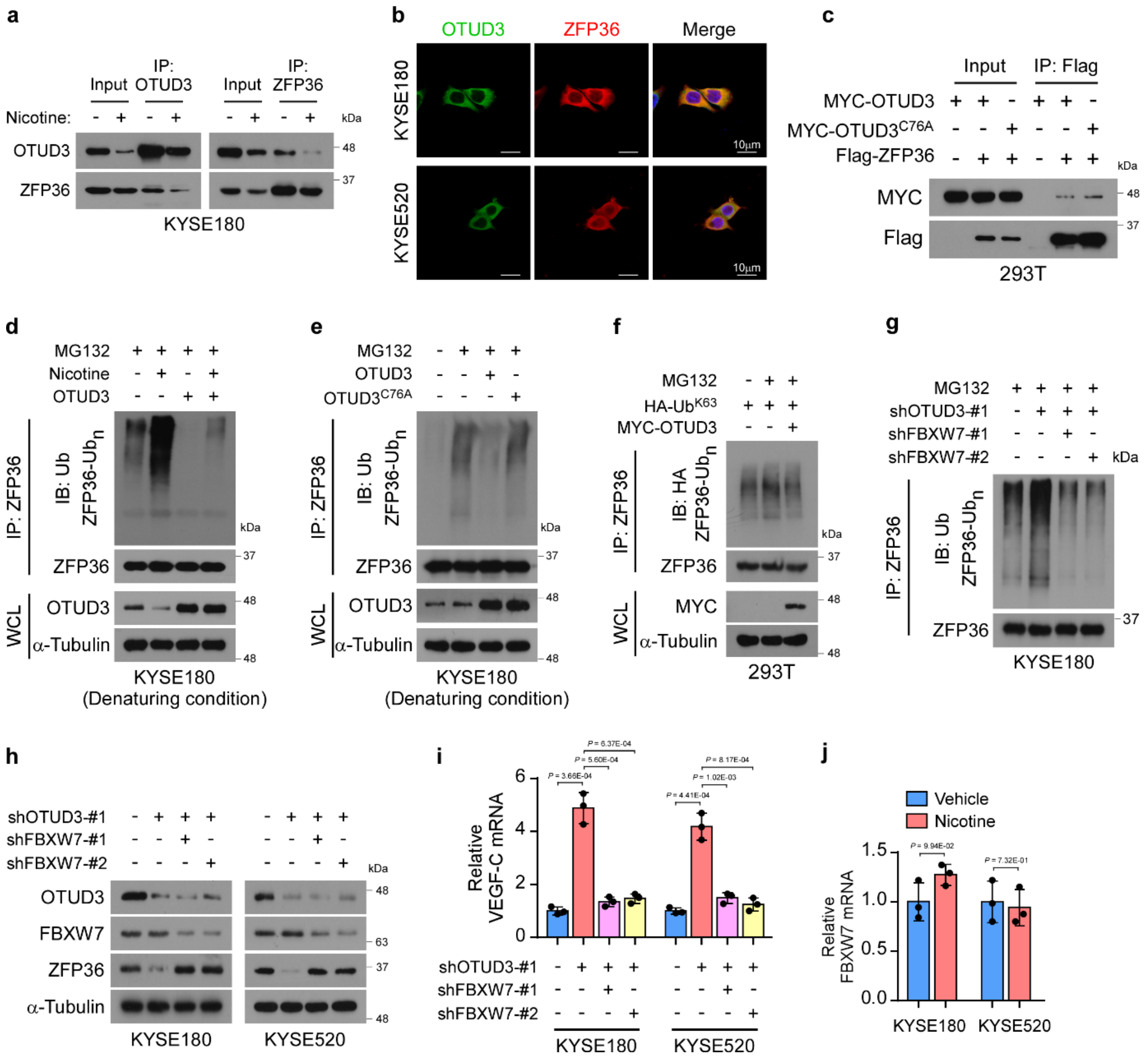

Supplementary Fig. 5. a Reciprocal IP assays of OTUD3 and ZFP36 were performed in KYSE180 cells with or without nicotine treatment. **b** Dual-IF staining of OTUD3 and ZFP36 in KYSE180 and KYSE520 cells. **c** Interactions between Flag-ZFP36 and MYC-OTUD3 or MYC-OTUD3^{C76A} were examined in 293T cells. **d** Vector or OTUD3-overexpressing KYSE180 cells were treated with or without nicotine. Cells were treated with MG132 (10 μM) for 6 h before harvest. Lysates were collected in denaturing buffer, followed by ZFP36 ubiquitination analysis. **e** Indicated KYSE180 cells were harvested in denaturing buffer, followed by ZFP36 ubiquitination analysis. **f** The 293T cells were transfected with HA-Ub^{K63} and MYC-OTUD3. Lysates were then subjected to ZFP36 ubiquitination analysis. **g** ZFP36 ubiquitination was examined in OTUD3-silencing KYSE180 cells with or without FBXW7 depletion. **h** Western blot analysis of indicated protein in OTUD3-silencing KYSE180 and KYSE520 cells with or without FBXW7 depletion. **i** qRT-PCR analysis of VEGF-C in indicated KYSE180 and KYSE520 cells. **j** qRT-PCR analysis of FBXW7 in KYSE180 and KYSE520 cells with vehicle or nicotine treatment. Each error bar in panels **i** and **j** represents the mean±SD of three biological replicates. Two-sided Student's t-test was used for statistical analysis. Source data are provided as a Source Data file.

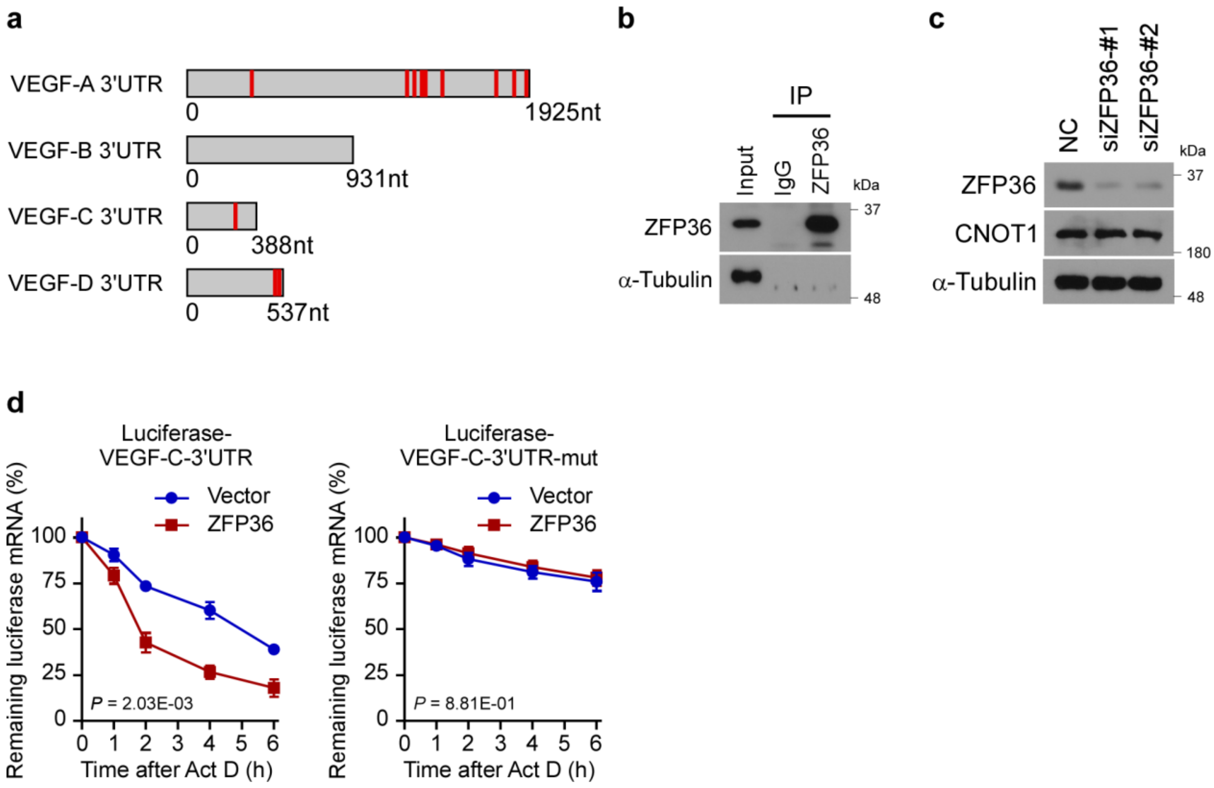

Supplementary Fig. 6. a A scheme showed the AREs in the 3'UTRs of VEGF family genes. **b** Immunoprecipitation of ZFP36 protein in KYSE180 cells was examined by western blot. **c** Western blot analysis of ZFP36 and CNOT1 in KYSE180 cells transfected with NC and ZFP36 siRNAs. **d** Vector or ZFP36-overexpressing KYSE180 cells were transfected with VEGF-C 3'UTR or 3'UTR-mut luciferase constructs then subjected to analysis of luciferase mRNA at indicated time points. One-way repeated-measures ANOVA test was used for statical analysis. Each error bar represents the mean±SD of three biological replicates. Source data are provided as a Source Data file.

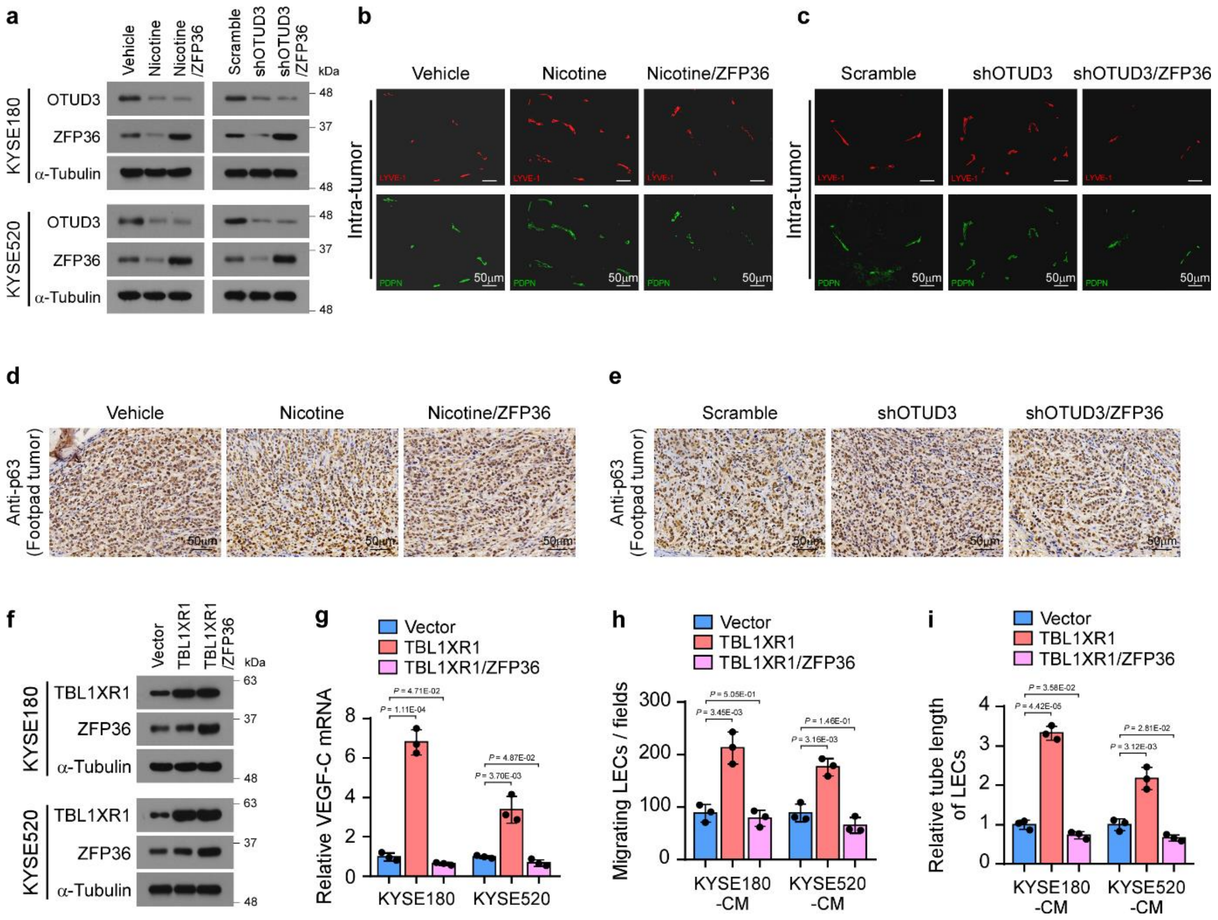

Supplementary Fig. 7. **a** Western blot analysis of OTUD3 and ZFP36 expression in indicated KYSE180 and KYSE520 cells. **b-c** Representative IF staining images of LYVE-1 and podoplanin (PDPN) in indicated footpad tumors. **d-e** Representative IHC staining of p63 in primary footpad tumors. **f** Western blot analysis of TBL1XR1 and ZFP36 expression in indicated KYSE180 and KYSE520 cells. **g** qRT-PCR analysis of VEGF-C mRNA expression in indicated esophageal cancer cells. **h** Quantification of migrating LECs under treatment of indicated CM. **i** Relative tube length of LECs under treatment of indicated CM. Each error bar in panels **g-i** represents the mean±SD of three biological replicates. Two-sided Student's t-test was used for statistical analysis. Source data are provided as a Source Data file.

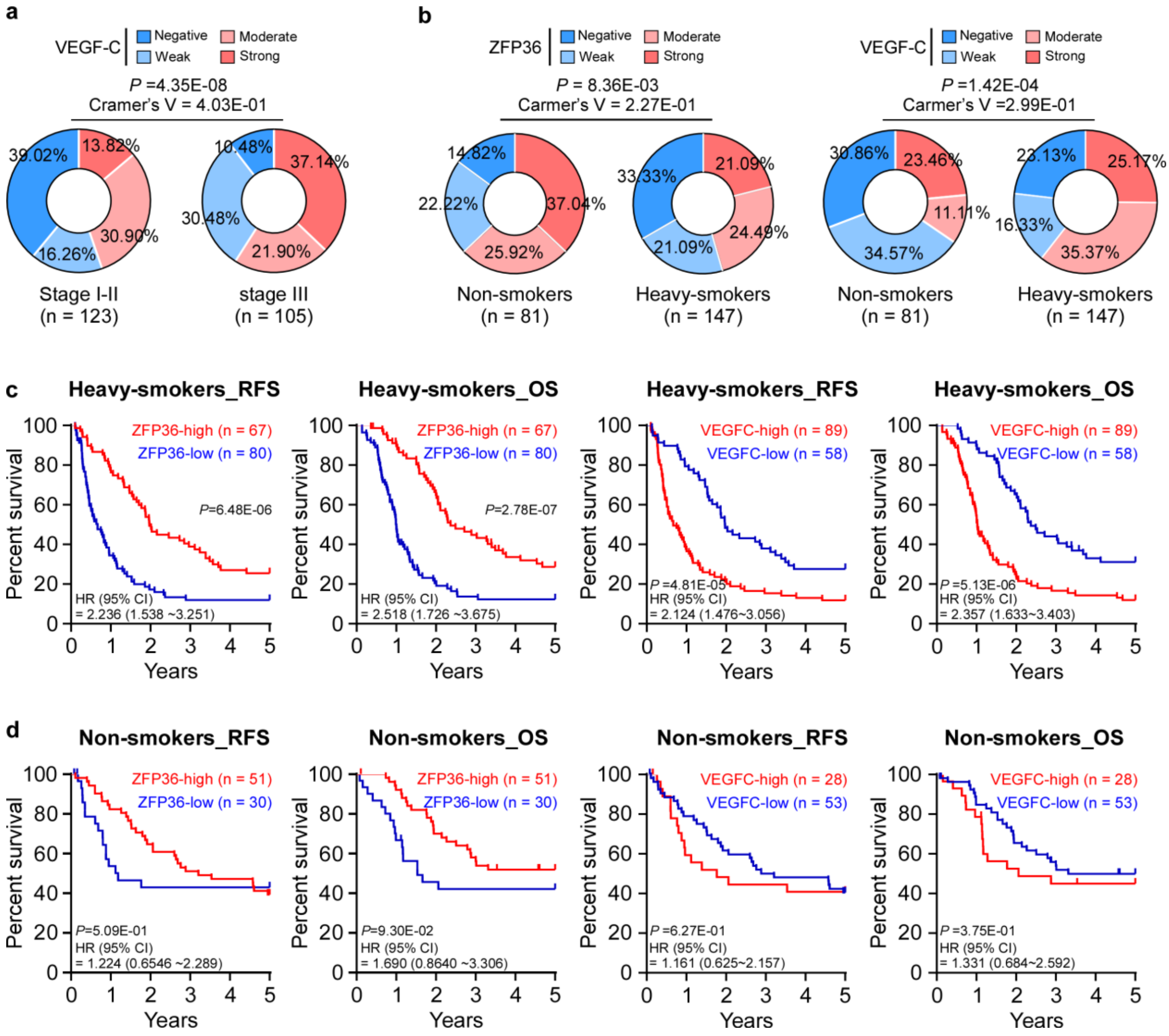

Supplementary Fig. 8. a The correlation between VEGF-C expression and clinical stages was analyzed. Two-sided χ^2 test and Cramer's V were used to evaluate the correlation. **b** The distributions of ZFP36 and VEGF-C expression in non-smoking and heavy-smoking esophageal cancer patients were compared. Two-sided χ^2 test and Cramer's V were used to test the correlation. **c-d** RFS and OS analyses in heavy-smoking (c) or non-smoking (d) esophageal cancer patients stratified by ZFP36 or VEGF-C expression. Log-rank test was used for statistical analysis. Source data are provided as a Source Data file.

Supplementary Fig. 9 Unprocessed scans of immunoblots shown in the figures

Fig. 1

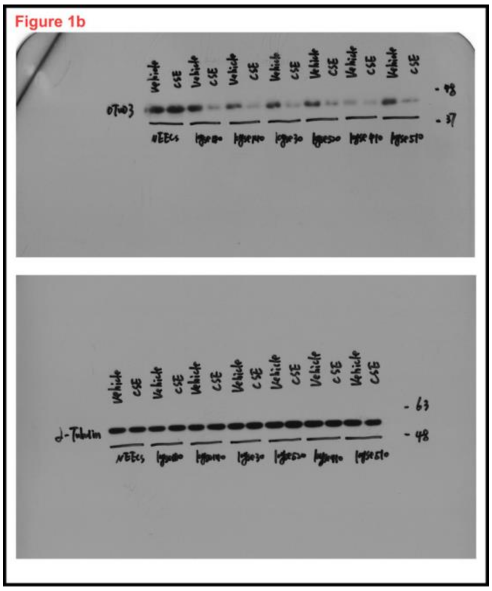

Fig. 2

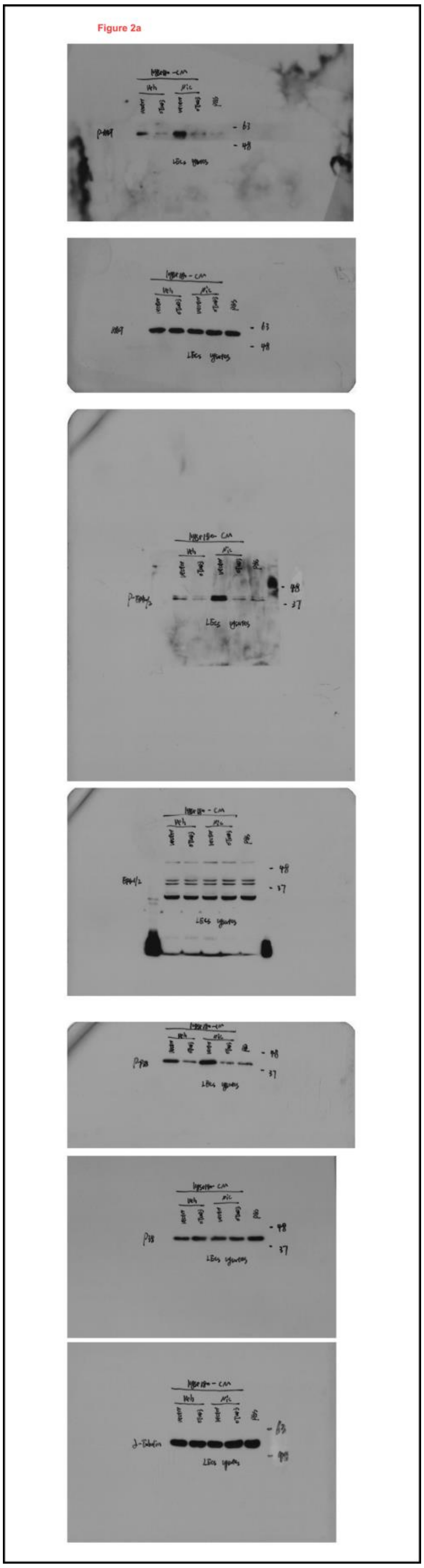

Fig. 3

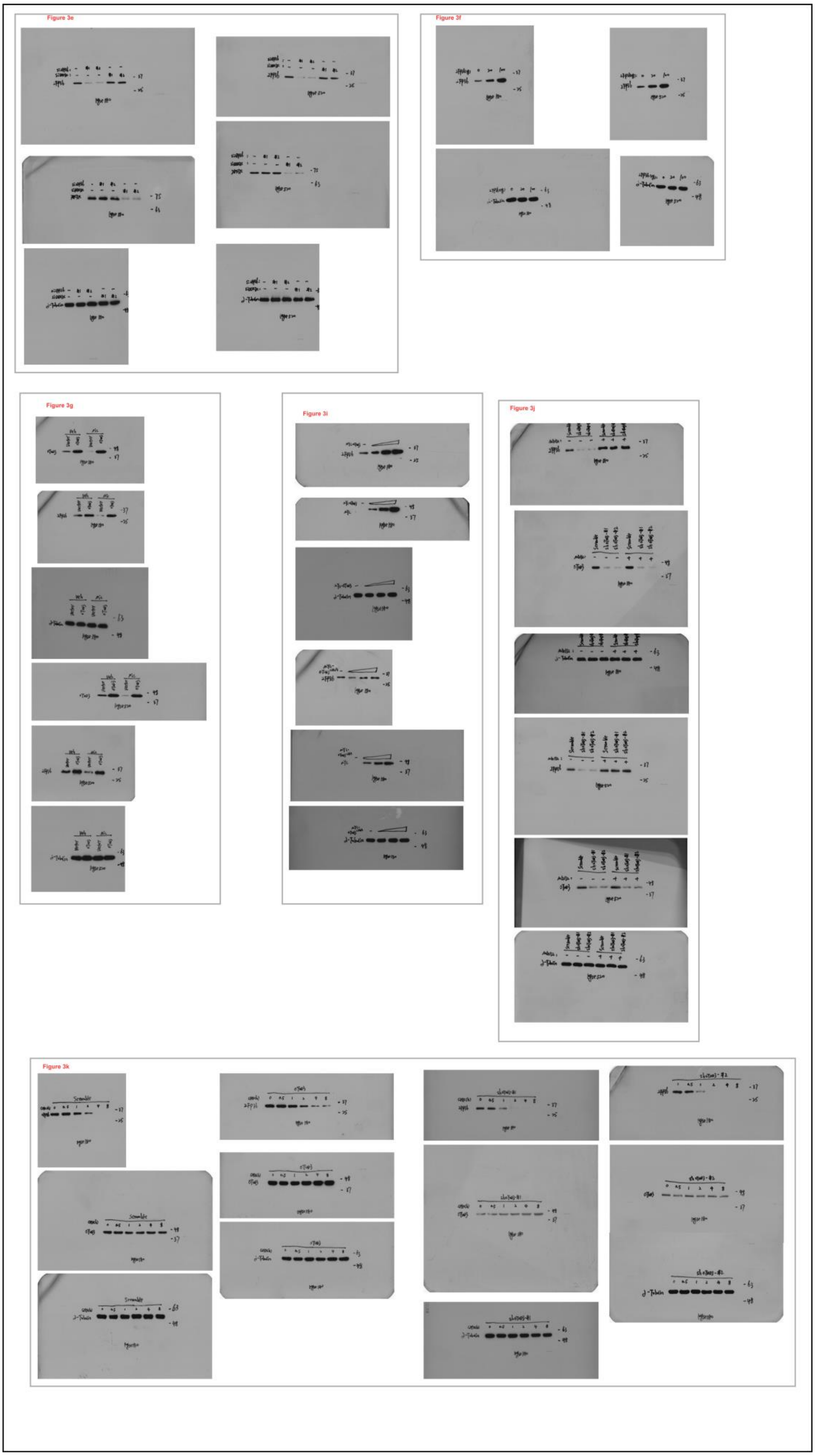

Fig. 4

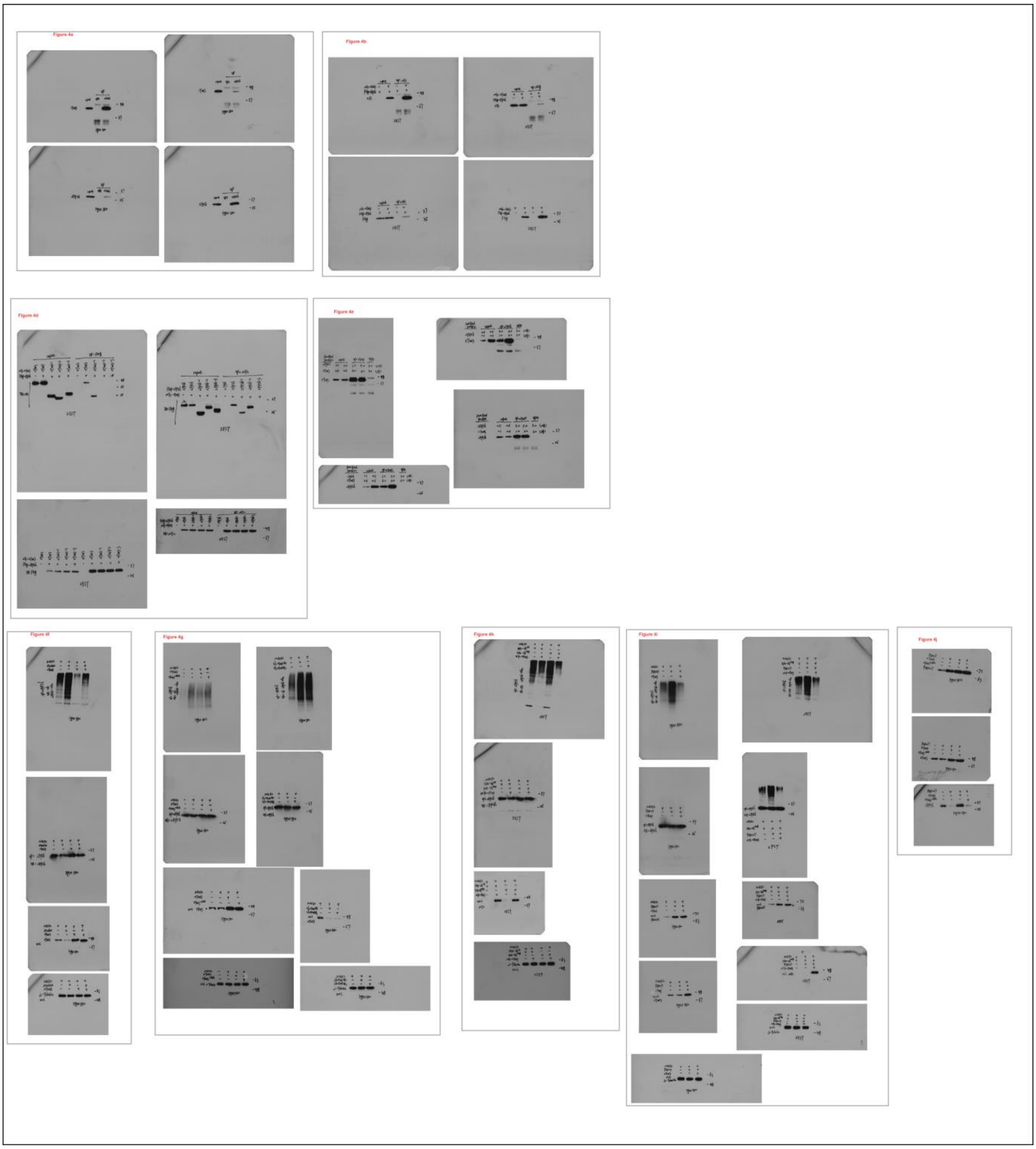
Figure 4a
Figure 4b
Figure 4d
Figure 4e
Figure 4f
Figure 4g
Figure 4h
Figure 4i
Figure 4j

Fig. 5

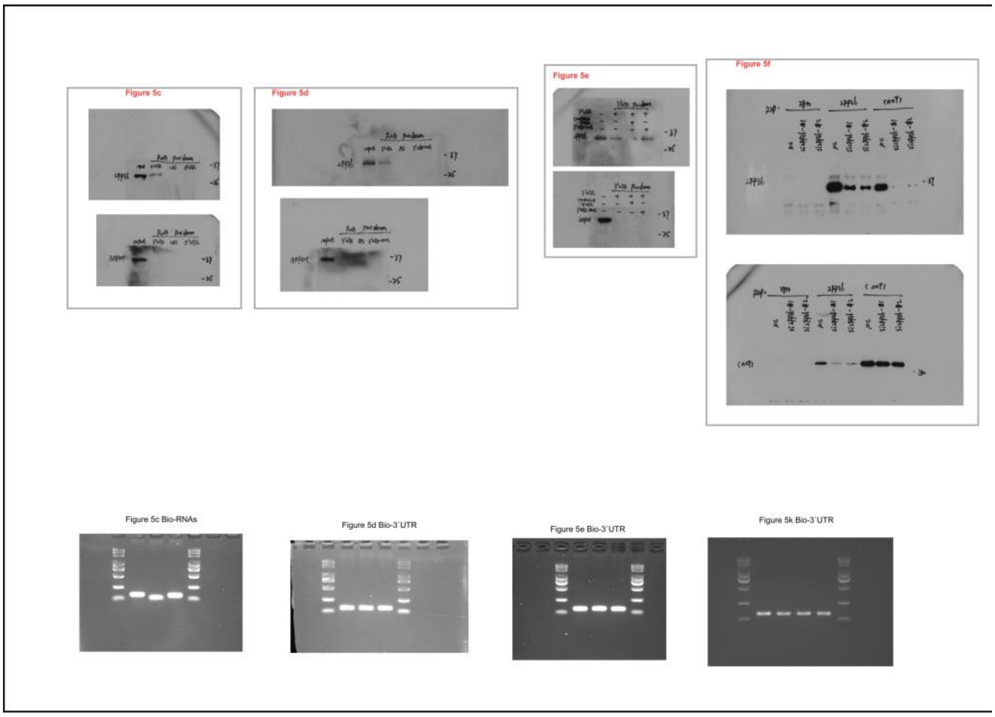
Figure 5c
Figure 5d
Figure 5e
Figure 5f
Figure 5c Bio-RNAs
Figure 5d Bio-3'UTR
Figure 5e Bio-3'UTR
Figure 5k Bio-3'UTR

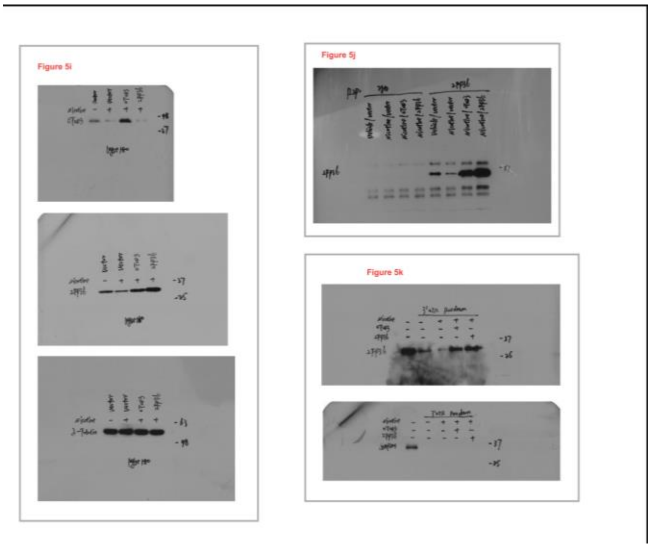
Figure 5i
Figure 5j
Figure 5k

Fig. 7

Figure 7a

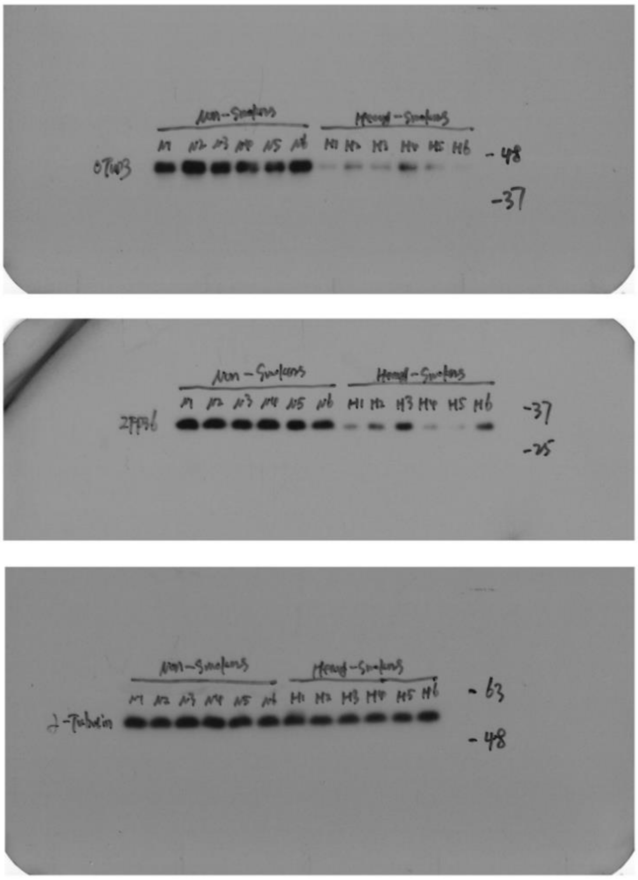

Supplementary Fig. 1

Supplementary Figure 1b

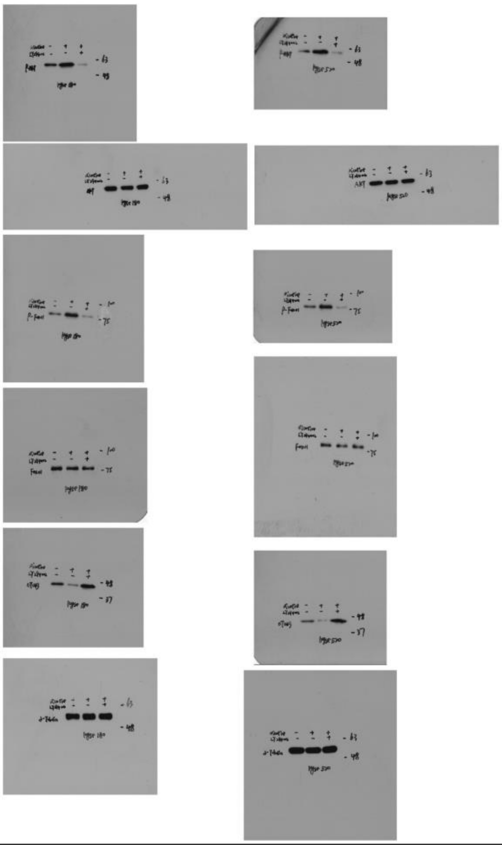

Supplementary Figure 1h

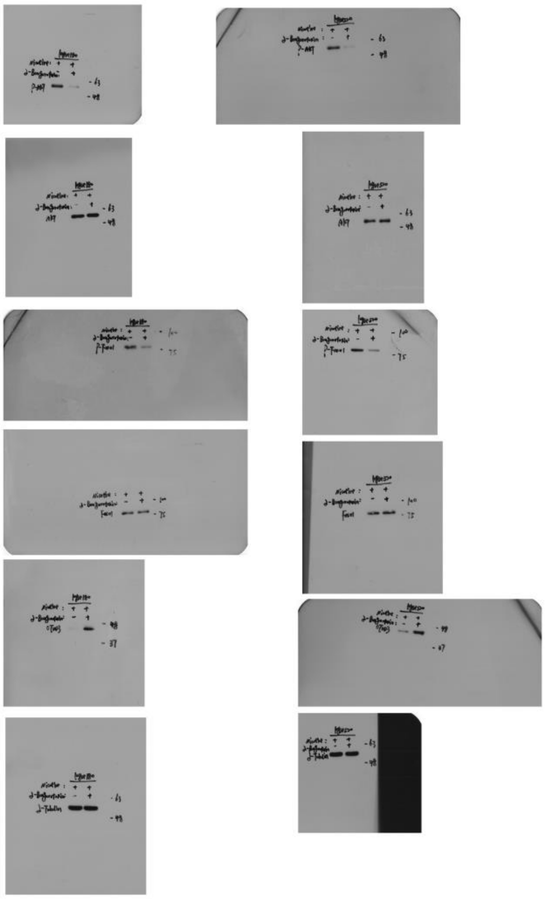

Supplementary Fig. 3

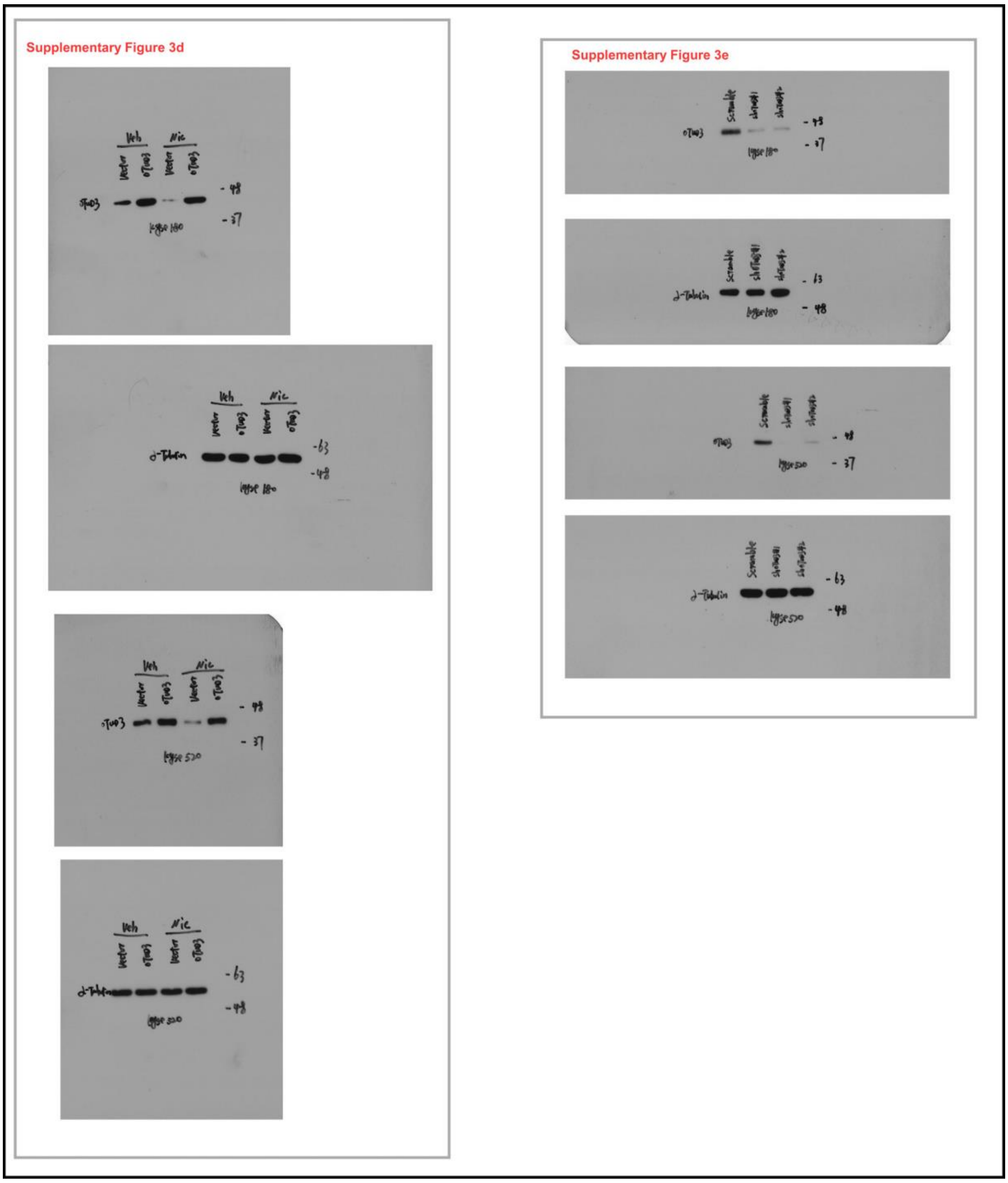

Supplementary Fig. 4

Supplementary Figure 4h

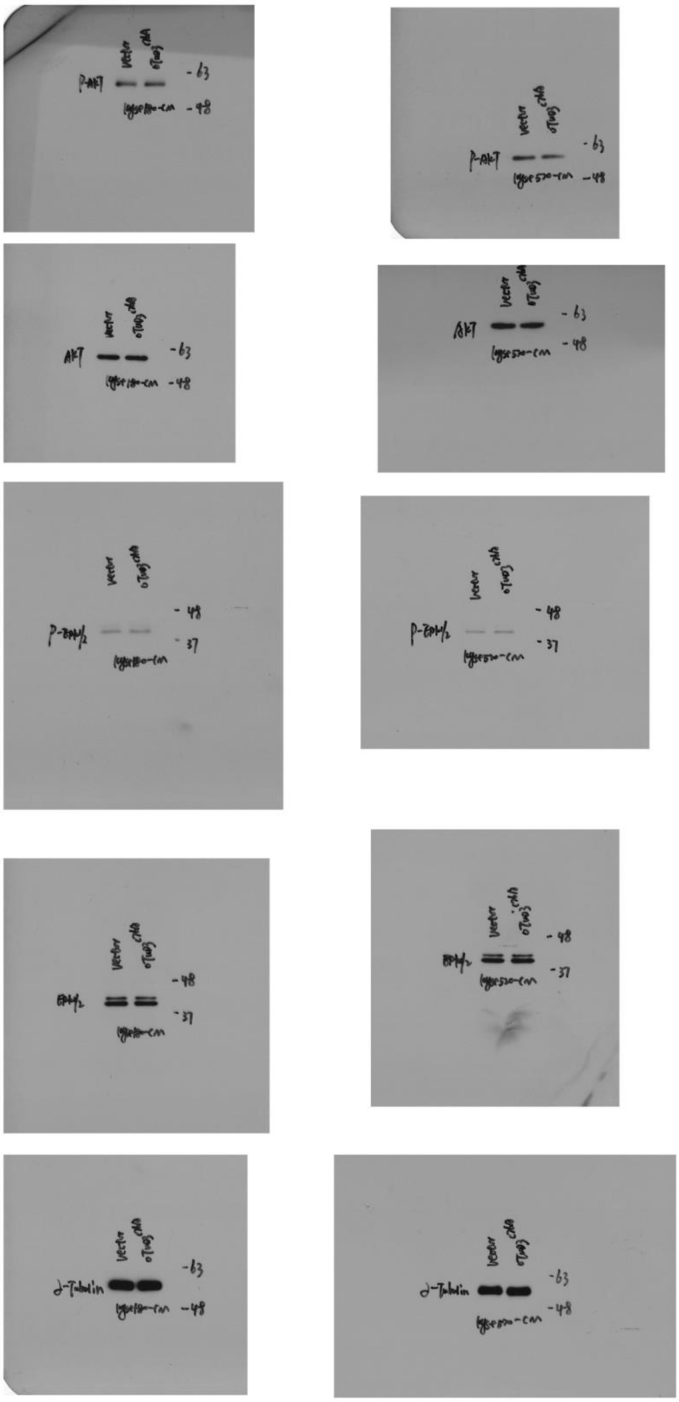

Supplementary Fig. 5

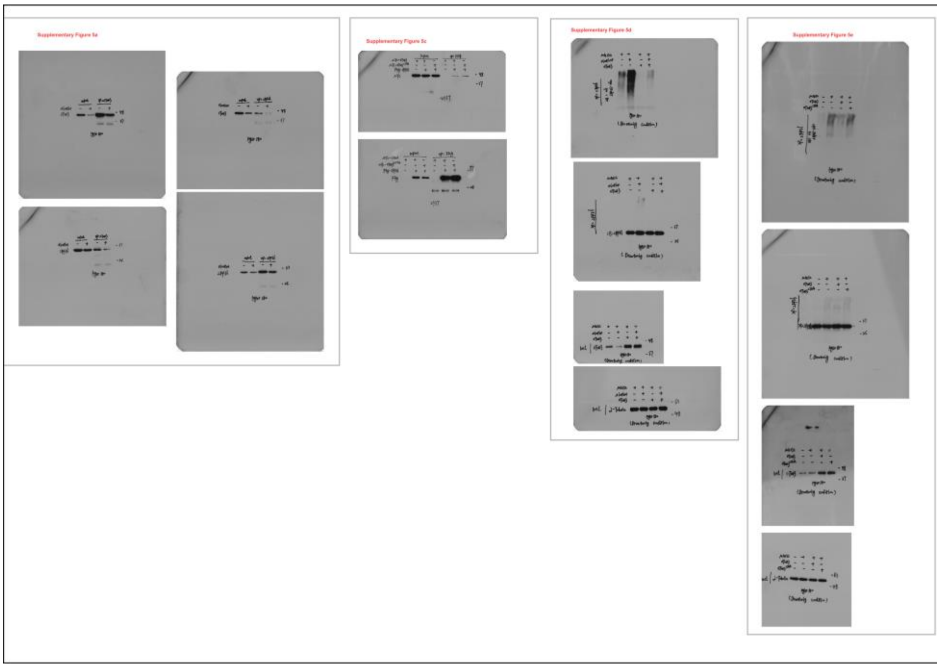

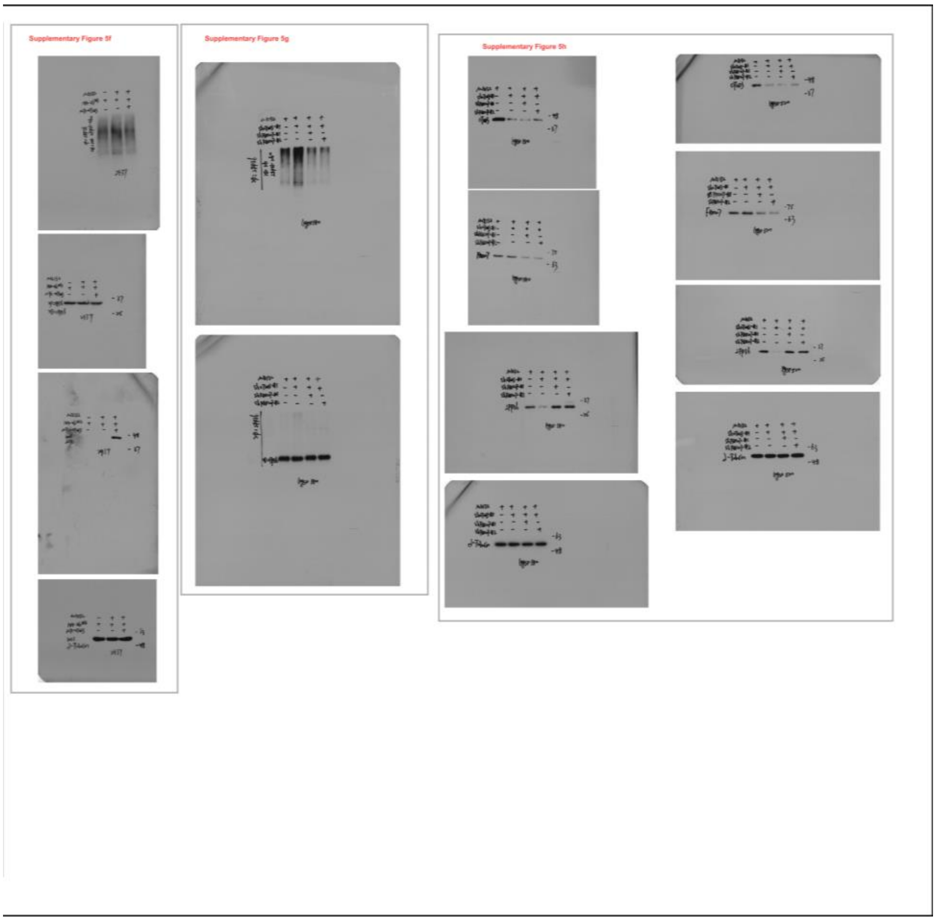

Supplementary Fig. 6

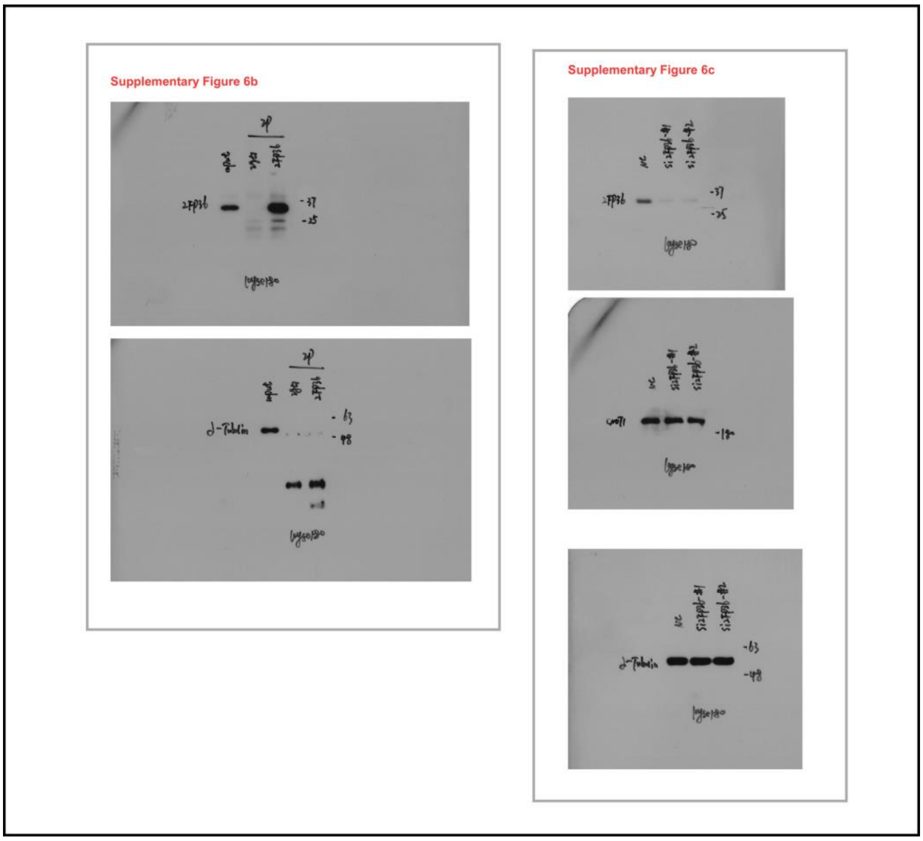

Supplementary Fig. 7

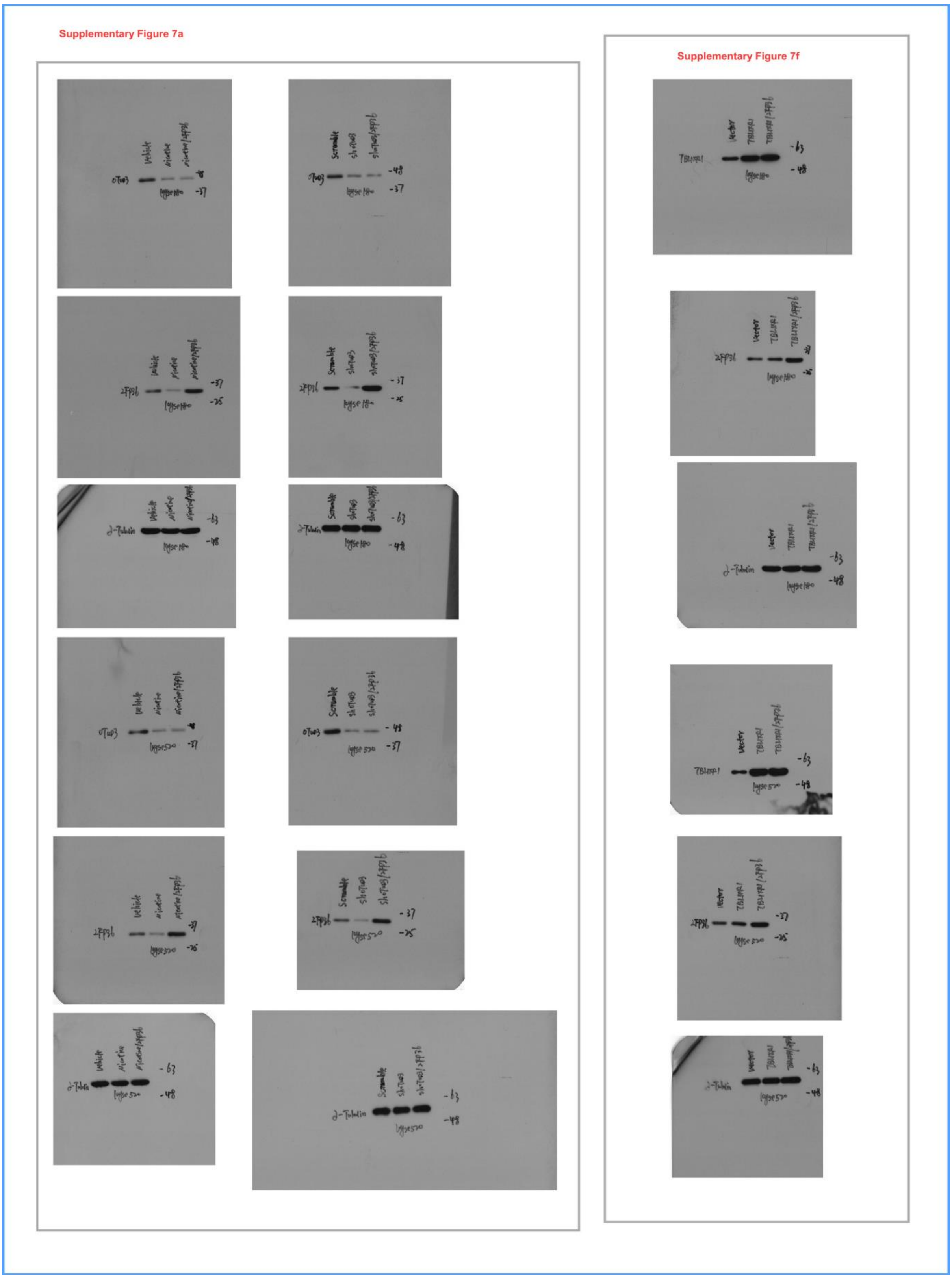

Supplementary Table 1. Clinicopathological characteristics of 228 esophageal cancer specimens

**Parameters**	**Number of cases (%)**
**Pathological type**	
Squamous cell carcinoma	228 (100.00%)
**Gender**	
Female	71 (31.14%)
Male	157 (68.86%)
**Age (years)**	
< 58	117 (51.32%)
≥ 58	111 (48.68%)
**T classification**	
T1	15 (6.58%)
T2	50 (21.93%)
T3	155 (67.98%)
T4	8 (3.51%)
**N classification**	
LN-	102 (44.74%)
LN+	126 (55.26%)
**Clinical stage**	
I	11 (4.82%)
II	112 (49.12%)
III	105 (46.05%)
**Pathological grade**	
G1	94 (41.23%)
G2	114 (50.00%)
G3	20 (8.77%)
**Smoking**	
Non-smokers	81 (35.53%)
Heavy-smokers	147 (64.47%)
**Smoking status**	
Non-smokers	81 (35.53%)
Formers	39 (17.10%)
Currents	108 (47.37%)
**Drinking**	
No	129 (56.58%)
Yes	99 (43.42%)
**Adjuvant therapy**	
No	18 (7.89%)
Yes	210 (92.11%)
**5-year tumor relapse status**	
No	64 (28.07%)
Yes	164 (71.93%)
**5-year vital status**	
Alive	72 (31.58%)
Dead	156 (68.42%)
**Nuc FOXO1 expression**	
Negative	90 (39.47%)
Positive	138 (60.53%)
**OTUD3 expression**	
Negative (0)	77 (33.77%)
Weak (1)	67 (29.39%)
Moderate (2)	45 (19.74%)
Strong (3)	39 (17.10%)
**ZFP36 expression**	
Negative (0)	61 (26.75%)
Weak (1)	49 (21.49%)
Moderate (2)	57 (25.00%)
Strong (3)	61 (26.75%)
**VEGF-C expression**	
Negative (0)	59 (25.88%)
Weak (1)	52 (22.81%)
Moderate (2)	61 (26.75%)
Strong (3)	56 (24.56%)

Supplementary Table 2. The correlation between OTUD3 expression and clinicopathological characteristics in esophageal cancer patients (n = 228)

Characteristics	OTUD3 expression<br>Low,<br>no. cases (%)	High,<br>no. cases (%)	P values<br>(Two-sided $\chi 2$ test)
**Gender**			
Male	109 (69.43%)	48 (30.57%)	0.004
Female	35 (49.30%)	36 (50.70%)	
**Age (years)**			
< 58	82 (70.09%)	35 (29.91%)	0.026
≥ 58	62 (55.86%)	49 (44.14%)	
**T stage**			
T1-2	29 (44.62%)	36 (55.38%)	< 0.001
T3-4	115 (70.55%)	48 (29.45%)	
**N stage**			
N0	53 (51.96%)	49 (48.04%)	0.002
N1	91 (72.22%)	35 (27.78%)	
**Clinical stage**			
I-II	63 (51.22%)	60 (48.78%)	< 0.001
III	81 (77.14%)	24 (22.86%)	
**Grade**			
G1	52 (55.32%)	42 (44.68%)	0.04
G2-3	92 (68.66%)	42 (31.34%)	
**Smoking**			
Non-smokers	33 (40.74%)	48 (59.26%)	< 0.001
Heavy-smokers	111 (75.51%)	36 (24.49%)	
**Drinking**			
No	81 (62.79%)	48 (37.21%)	0.896
Yes	63 (63.64%)	36 (36.36%)	
**5-year tumor relapse status**			
No	30 (46.88%)	34 (53.12%)	0.001
Yes	114 (69.51%)	50 (30.49%)	
**5-year vital status**			
Alive	34 (47.22%)	38 (52.78%)	< 0.001
Dead	110 (70.51%)	46 (29.49%)	
**Nuc FOXO1 expression**			
Negative	129 (93.48%)	9 (6.51%)	< 0.001
Positive	15 (16.67%)	75 (83.33%)	
**ZFP36 expression**			
Negative (0)	54 (88.52%)	7 (11.48%)	< 0.001
Weak (1)	32 (65.31%)	17 (34.69%)	
Moderate (2)	36 (63.16%)	21 (36.84%)	
Strong (3)	22 (36.06%)	39 (63.93%)	
**VEGF-C expression**			
Negative (0)	19 (32.20%)	40 (67.80%)	< 0.001
Weak (1)	40 (76.92%)	12 (23.08%)	
Moderate (2)	35 (57.38%)	26 (42.62%)	
Strong (3)	50 (89.29%)	6 (10.71%)	

Supplementary Table 3. The correlation between nuclear FOXO1 expression and clinicopathological characteristics in esophageal cancer patients (n = 228)

	**Nuc FOXO1 expression**		
Characteristics	Negative,	Positive,	P values (Two-sided χ2 test)
	no. cases (%)	no. cases (%)	
**Gender**			
Male	111 (70.70%)	46 (29.30%)	< 0.001
Female	27 (38.03%)	44 (61.97%)	
**Age (years)**			
< 58	76 (64.96%)	41 (35.04%)	0.16
≥ 58	62 (55.86%)	49 (44.14%)	
**T stage**			
T1-2	27 (41.54%)	38 (58.46%)	< 0.001
T3-4	111 (68.10%)	52 (31.90%)	
**N stage**			
N0	54 (52.94%)	48 (47.06%)	0.035
N1	84 (66.67%)	42 (33.33%)	
**Clinical stage**			
I-II	63 (51.22%)	60 (48.78%)	0.002
III	75 (71.43%)	30 (28.57%)	
**Grade**			
G1	53 (56.38%)	41 (43.62%)	0.284
G2-3	85 (63.43%)	49 (36.57%)	
**Smoking**			
Non-smokers	31 (38.27%)	50 (61.73%)	< 0.001
Heavy-smokers	107 (72.79%)	40 (27.21%)	
**Drinking**			
No	73 (56.59%)	56 (43.41%)	0.165
Yes	65 (65.66%)	34 (34.34%)	
**5-year tumor relapse status**			
No	31 (48.44%)	33 (51.56%)	0.02
Yes	107 (65.24%)	57 (34.75%)	
**5-year vital status**			
Alive	35 (48.61%)	37 (51.39%)	0.012
Dead	103 (66.03%)	53 (33.97%)	
**ZFP36 expression**			
Negative (0)	47 (77.05%)	14 (22.95%)	0.001
Weak (1)	33 (67.35%)	16 (32.65%)	
Moderate (2)	31 (54.39%)	26 (45.61%)	
Strong (3)	27 (44.26%)	34 (55.74%)	
**VEGF-C expression**			
Negative (0)	23 (38.98%)	36 (61.02%)	< 0.001
Weak (1)	31 (59.62%)	21 (40.38%)	
Moderate (2)	38 (62.30%)	23 (37.70%)	
Strong (3)	46 (82.14%)	10 (17.86%)	

Supplementary Table 4. The correlation between ZFP36 expression and clinicopathological characteristics in esophageal cancer patients (n = 228)

	ZFP36 expression		
Characteristics	Low,	High,	P values (Two-sided χ2 test)
	no. cases (%)	no. cases (%)	
**Gender**			
Male	80 (50.96%)	77 (49.04%)	0.223
Female	30 (42.25%)	41 (57.75%)	
**Age (years)**			
< 58	63 (53.85%)	54 (46.15%)	0.082
≥ 58	47 (42.34%)	64 (57.66%)	
**T stage**			
T1-2	25 (38.46%)	40 (61.54%)	0.062
T3-4	85 (52.14%)	78 (47.85%)	
**N stage**			
N0	40 (39.22%)	62 (60.78%)	0.014
N1	70 (55.56%)	56 (44.44%)	
**Clinical stage**			
I-II	50 (40.65%)	73 (59.35%)	0.013
III	60 (57.14%)	45 (42.86%)	
**Grade**			
G1	44 (46.81%)	50 (53.19%)	0.716
G2-3	66 (49.25%)	68 (50.75%)	
**Smoking**			
Non-smokers	30 (37.04%)	51 (62.96%)	0.012
Heavy-smokers	80 (54.42%)	67 (45.58%)	
**Drinking**			
No	57 (44.19%)	72 (55.81%)	0.161
Yes	53 (53.54%)	46 (46.46%)	
**5-year tumor relapse status**			
No	27 (42.19%)	37 (57.81%)	0.253
Yes	83 (49.39%)	81 (50.61%)	
**5-year vital status**			
Alive	24 (33.33%)	48 (66.67%)	0.002
Dead	86 (55.13%)	70 (44.87%)	
**VEGF-C expression**			
Negative (0)	14 (23.73%)	45 (76.27%)	< 0.001
Weak (1)	20 (38.46%)	32 (61.54%)	
Moderate (2)	36 (59.02%)	25 (40.98%)	
Strong (3)	40 (71.43%)	16 (28.57%)	

Supplementary Table 5. The correlation between VEGF-C expression and clinicopathological characteristics in esophageal cancer patients (n = 228)

	**VEGF-C expression**		
Characteristics	Low,	High,	P values (Two-sided χ2 test)
	no. cases (%)	no. cases (%)	
**Gender**			
Male	61 (38.85%)	96 (61.15%)	< 0.001
Female	50 (70.42%)	21 (29.58%)	
**Age (years)**			
< 58	61 (52.14%)	56 (47.86%)	0.284
≥ 58	50 (45.05%)	61 (54.95%)	
**T stage**			
T1-2	41 (63.08%)	24 (36.92%)	0.006
T3-4	70 (42.94%)	93 (57.06%)	
**N stage**			
N0	63 (61.76%)	39 (38.24%)	< 0.001
N1	48 (38.10%)	78 (61.90%)	
**Clinical stage**			
I-II	68 (55.28%)	55 (44.71%)	0.031
III	43 (40.95%)	62 (59.05%)	
**Grade**			
G1	49 (52.13%)	45 (47.87%)	0.384
G2-3	62 (46.27%)	72 (53.73%)	
**Smoking**			
Non-smokers	53 (65.43%)	28 (34.57%)	< 0.001
Heavy-smokers	58 (39.46%)	89 (60.54%)	
**Drinking**			
No	72 (55.81%)	57 (44.19%)	0.014
Yes	39 (39.39%)	60 (60.61%)	
**5-year tumor relapse status**			
No	38 (59.38%)	26 (40.63%)	0.044
Yes	73 (44.51%)	91 (55.49%)	
**5-year vital status**			
Alive	46 (63.89%)	26 (36.11%)	0.002
Dead	65 (41.67%)	91 (58.33%)	

Supplementary Table 6. Antibodies with the applications in which they were used

Name/Assay	Catalogue No.	Company	Concentration	Source
**IHC**				
anti-OTUD3	HPA028544	Sigma-Aldrich	1:100	rabbit
anti-LYVE1	HPA042953	Sigma-Aldrich	1:200	rabbit
anti-p63	SAB5600140	Sigma-Aldrich	1:1000	rabbit
anti-ZFP36	ABE285	Merck Millipore	1:50	rabbit
anti-VEGFC	AF752	R&D	1:50	goat
anti-FOXO1	#2880	CST	1:100	rabbit
**WB**				
anti-p-AKT	#4060	CST	1:2000	rabbit
anti-AKT	#9272	CST	1:1000	rabbit
anti-p-ERK1/2	#4370	CST	1:2000	rabbit
anti-ERK1/2	#4695	CST	1:1000	rabbit
anti-p-p38	#4511	CST	1:1000	rabbit
anti-p38	#8690	CST	1:1000	rabbit
anti-p-FOXO1	#9464	CST	1:1000	rabbit
anti-FOXO1	#2880	CST	1:1000	rabbit
anti-α-Tubulin	T9026	Sigma-Aldrich	1:500	mouse
anti-ZFP36	ABE285	Merck Millipore	1:1000	rabbit
anti-OTUD3	MABS1819M	Sigma-Aldrich	1:500	mouse
anti-MYC	#13987	CST	1:1000	rabbit
anti-Flag	F7425	Sigma-Aldrich	1:1000	rabbit
anti-Ubiquitin	#3936	CST	1:1000	mouse
anti-HA	#3724	CST	1:1000	rabbit
anti-FBXW7	ab109617	Abcam	1:1000	rabbit
anti-GAPDH	#5174	CST	1:1000	rabbit
anti-TBL1XR1	ab190796	Abcam	1:1000	rabbit
**IF**				
anti-OTUD3	HPA028543	Sigma-Aldrich	1:100	rabbit
anti-ZFP36	ab124024	Abcam	1:100	mouse
anti-PDPN	ab10288	Abcam	1:500	mouse
anti-LYVE1	#67538	CST	1:200	rabbit
**PLA**				
anti-OTUD3	HPA028544	Sigma-Aldrich	1:100	rabbit
anti-ZFP36	ab124024	Abcam	1:100	mouse
**IP**				
anti-OTUD3	MABS1819M	Sigma-Aldrich		mouse
anti-Flag	F7425	Sigma-Aldrich		rabbit
anti-ZFP36	ABE285	Merck Millipore		rabbit
anti-MYC	#2276	CST		mouse
**RIP**				
anti-ZFP36	ABE285	Merck Millipore		rabbit
anti-CNOT1	A305-787A	Bethyl		rabbit
anti-IgG	ab172730	Abcam		rabbit
**ChIP**				
anti-FOXO1	#2880	CST		rabbit
anti-p300	ab275378	Abcam		rabbit
anti-H3K27ac	ab4729	Abcam		rabbit
anti-Polymerase II	ab264350	Abcam		rabbit
anti-IgG	ab172730	Abcam		rabbit
