# Supplementary material for: Nicotine-mediated OTUD3 downregulation inhibits VEGF-C mRNA decay to promote lymphatic metastasis of human esophageal cancer
Source: Nat Commun. 2021 Dec 1;12:7006. doi: 10.1038/s41467-021-27348-8 (PMC8636640; doi:10.1038/s41467-021-27348-8)
Supplement: Supplementary file 3 — Description of Additional Supplementary Files [file 41467_2021_27348_MOESM3_ESM.pdf]

Description of Additional Supplementary Files

Supplementary Data 1. A table shows peptides and counts for OTUD3-binding proteins analyzed by immunoprecipitation (IP)/mass spectrometry (MS) assays

Supplementary Data 2. Information on primers and oligonucleotides used in this study
