# Supplementary material for: Nicotine-mediated OTUD3 downregulation inhibits VEGF-C mRNA decay to promote lymphatic metastasis of human esophageal cancer
Source: Nat Commun. 2021 Dec 1;12:7006. doi: 10.1038/s41467-021-27348-8 (PMC8636640; doi:10.1038/s41467-021-27348-8)
Supplement: Supplementary file 6 — Reporting Summary [file 41467_2021_27348_MOESM6_ESM.pdf]

nature portfolio

Corresponding author(s): Libing Song

Last updated by author(s): Nov 5, 2021

Reporting Summary

Nature Portfolio wishes to improve the reproducibility of the work that we publish. This form provides structure for consistency and transparency in reporting. For further information on Nature Portfolio policies, see our Editorial Policies and the Editorial Policy Checklist.

Statistics

For all statistical analyses, confirm that the following items are present in the figure legend, table legend, main text, or Methods section.

n/a	Confirmed	
☐	☒	The exact sample size (*n*) for each experimental group/condition, given as a discrete number and unit of measurement
☐	☒	A statement on whether measurements were taken from distinct samples or whether the same sample was measured repeatedly
☐	☒	The statistical test(s) used AND whether they are one- or two-sided<br>*Only common tests should be described solely by name; describe more complex techniques in the Methods section.*
☒	☐	A description of all covariates tested
☒	☐	A description of any assumptions or corrections, such as tests of normality and adjustment for multiple comparisons
☐	☒	A full description of the statistical parameters including central tendency (e.g. means) or other basic estimates (e.g. regression coefficient) AND variation (e.g. standard deviation) or associated estimates of uncertainty (e.g. confidence intervals)
☐	☒	For null hypothesis testing, the test statistic (e.g. *F*, *t*, *r*) with confidence intervals, effect sizes, degrees of freedom and *P* value noted<br>*Give P values as exact values whenever suitable.*
☒	☐	For Bayesian analysis, information on the choice of priors and Markov chain Monte Carlo settings
☒	☐	For hierarchical and complex designs, identification of the appropriate level for tests and full reporting of outcomes
☒	☐	Estimates of effect sizes (e.g. Cohen's *d*, Pearson's *r*), indicating how they were calculated

Our web collection on statistics for biologists contains articles on many of the points above.

Software and code

Policy information about availability of computer code

Data collection	Fluorescent staining images were obtained by using laser scanning confocal microscopy (LSM880, Carl Zeiss MicroImaging, Oberkochen, Germany);The iamges of the human lymphatic endothelial cells (LECs) migration and tube formation assays were viewed using a microscope (Eclipse 80i, Nikon, Tokyo, Japan).
Data analysis	Statistical analyses are performed using the SPSS version 19.0 statistical software package and Graph-Pad Prism 8 version 8.3.0 software (GraphPad software, La Jolla, CA, USA).Western blot grayscale analyses were performed using image J 1.42q software.

For manuscripts utilizing custom algorithms or software that are central to the research but not yet described in published literature, software must be made available to editors and reviewers. We strongly encourage code deposition in a community repository (e.g. GitHub). See the Nature Portfolio guidelines for submitting code & software for further information.

Data

Policy information about availability of data

All manuscripts must include a data availability statement. This statement should provide the following information, where applicable:

- Accession codes, unique identifiers, or web links for publicly available datasets
- A description of any restrictions on data availability
- For clinical datasets or third party data, please ensure that the statement adheres to our policy

The RNA-seq data have been deposited in the National Center for Biotechnology Information Sequence Read Archive (SRA) database (https://www.ncbi.nlm.nih.gov/sra/PRJNA678868). The mass spectrometry proteomics data have been deposited to the ProteomeXchange Consortium (http://proteomecentral.proteomexchange.org) via the iProX partner repository with the dataset identifier PXD028751. Source data are provided with this paper.All the other data supporting the findings of this study are available within the article and its Supplementary information files. A reporting summary for this article is

available as a Supplementary Information file.

Field-specific reporting

Please select the one below that is the best fit for your research. If you are not sure, read the appropriate sections before making your selection.

- [x] Life sciences
- [ ] Behavioural & social sciences
- [ ] Ecological, evolutionary & environmental sciences

For a reference copy of the document with all sections, see nature.com/documents/nr-reporting-summary-flat.pdf

Life sciences study design

All studies must disclose on these points even when the disclosure is negative.

Sample size	Sample sizes for each experiment are stated in figure legends. For in vitro assays, n =3. For in vivo mouse experiments, usually n =6 mice were used for analyses. No statistical method was used to predetermine sample size.Sample sizes are determined empirically, and similar in size to most existing studies in the same field(PMCID: PMC8497616 ;PMCID: PMC8438024).
Data exclusions	No data were excluded from the analyses.
Replication	Both technical and biological replications were done. All attempts to reproduce data were successful. The number of repeats were indicated in figure legends.
Randomization	For all in vivo experiments,mice were randomly allocated among groups.For IHC,IF and PLA assays, 5 fields of view were randomly selected for each slice.
Blinding	For animal experiments, investigators were not blinded of group assignment during data collection and/or analysis since because gave different treatments among groups and measured the value of lymph nodes size(PMID: 34593796;PMID: 34475402). Since the mice were randomly allocated among groups, blinding should not be relevant in these analyses.The experimental results of molecular and cellular biology are obtained by objective quantitative methods(PMCID: PMC8438024), so we were not blinded to sample allocation.

Reporting for specific materials, systems and methods

We require information from authors about some types of materials, experimental systems and methods used in many studies. Here, indicate whether each material, system or method listed is relevant to your study. If you are not sure if a list item applies to your research, read the appropriate section before selecting a response.

Materials & experimental systems

n/a	Involved in the study	
[ ]	[x]	Antibodies
[ ]	[x]	Eukaryotic cell lines
[x]	[ ]	Palaeontology and archaeology
[ ]	[x]	Animals and other organisms
[ ]	[x]	Human research participants
[x]	[ ]	Clinical data
[x]	[ ]	Dual use research of concern

Methods

n/a	Involved in the study	
[x]	[ ]	ChIP-seq
[x]	[ ]	Flow cytometry
[x]	[ ]	MRI-based neuroimaging

Antibodies

Antibodies used	All antibodies have been described in Supplementary information.
Validation	Validation was relied on the available data on commercial websites.<br>The following antibodies have been approved by the manufacturers for IHC assay:<br>anti-OTUD3(HPA028544 /Sigma-Aldrich /rabbit):https://www.sigmaaldrich.cn/CN/zh/product/sigma/hpa028544?context=product<br>anti-LYVE1(HPA042953/Sigma-Aldrich/rabbit):https://www.sigmaaldrich.cn/CN/zh/product/sigma/hpa042953?context=product<br>anti-p63(SAB5600140/Sigma-Aldrich/rabbit):https://www.sigmaaldrich.cn/CN/zh/product/sigma/sab5600140?context=product<br>anti-ZFP36(ABE285/Merck Millipore/rabbit):https://www.sigmaaldrich.cn/CN/zh/product/mm/abe285?context=product √<br>anti-VEGFC (AF752/R&D/Goat):https://www.rndsystems.com/cn/products/human-vegf-c-antibody_af752<br>anti-FOXO1(#2880/ CST/rabbit):https://www.cellsignal.cn/products/primary-antibodies/foxo1-c29h4-rabbit-mab/2880?site-search-type=Products&N=4294956287&Ntt=%232880&fromPage=plp&_requestid=332406<br><br>The following antibodies have been approved by the manufacturers for WB assay:<br>anti-p-AKT(#4060/CST/rabbit):https://www.cellsignal.cn/products/primary-antibodies/phospho-akt-ser473-d9e-xp-rabbit-mab/4060?site-search-type=Products&N=4294956287&Ntt=%234060&fromPage=plp&_requestid=332504

anti-AKT(#9272/CST/rabbit) https://www.cellsignal.cn/products/primary-antibodies/akt-antibody/9272?site-search-type=Products&N=4294956287&Ntt=%239272&fromPage=plp&_requestid=332543
anti-p-ERK1/2(#4370/CST/rabbit):https://www.cellsignal.cn/products/primary-antibodies/phospho-p44-42-mapk-erk1-2-thr202-tyr204-d13-14-4e-xp-rabbit-mab/4370?site-search-type=Products&N=4294956287&Ntt=%234370&fromPage=plp&_requestid=332569
anti-ERK1/2(#4695 /CST/rabbit):https://www.cellsignal.cn/products/primary-antibodies/p44-42-mapk-erk1-2-137f5-rabbit-mab/4695?site-search-type=Products&N=4294956287&Ntt=%234695&fromPage=plp
anti-p-p38(#4511/CST/rabbit):https://www.cellsignal.cn/products/primary-antibodies/phospho-p38-mapk-thr180-tyr182-d3f9-xp-rabbit-mab/4511?site-search-type=Products&N=4294956287&Ntt=%234511&fromPage=plp&_requestid=332699
anti-p38(#8690/CST/rabbit):https://www.cellsignal.cn/products/primary-antibodies/p38-mapk-d13e1-xp-rabbit-mab/8690?site-search-type=Products&N=4294956287&Ntt=%238690&fromPage=plp&_requestid=332726
anti-p-FOXO1(#9464/CST/rabbit):https://www.cellsignal.cn/products/primary-antibodies/phospho-foxo1-thr24-foxo3a-thr32-antibody/9464?site-search-type=Products&N=4294956287&Ntt=%239464&fromPage=plp&_requestid=332770
anti-FOXO1(#2880/CST/rabbit):https://www.cellsignal.cn/products/primary-antibodies/foxo1-c29h4-rabbit-mab/2880?site-search-type=Products&N=4294956287&Ntt=%232880&fromPage=plp&_requestid=332406
anti-α-Tubulin(T9026/Sigma-Aldrich/mouse):https://www.sigmaaldrich.cn/CN/zh/product/sigma/t9026?context=product
anti-ZFP36(ABE285/Merck Millipore/rabbit):https://www.sigmaaldrich.cn/CN/zh/product/mm/abe285?context=product
anti-OTUD3(MABS1819M/Sigma-Aldrich/mouse):https://www.sigmaaldrich.cn/CN/zh/product/mm/mabs1819m?context=product
anti-MYC(#13987/CST/rabbit):https://www.cellsignal.cn/products/primary-antibodies/c-myc-n-myc-d3n8f-rabbit-mab/13987?site-search-type=Products&N=4294956287&Ntt=%2313987&fromPage=plp&_requestid=332952anti-Flag(F7425/Sigma-Aldrich/rabbit):
anti-Ubiquitin(#3936/CST/mouse):https://www.cellsignal.cn/products/primary-antibodies/ubiquitin-p4d1-mouse-mab/3936?site-search-type=Products&N=4294956287&Ntt=%233936&fromPage=plp&_requestid=332990
anti-HA(#3724/CST/rabbit):https://www.cellsignal.cn/products/primary-antibodies/ha-tag-c29f4-rabbit-mab/3724?site-search-type=Products&N=4294956287&Ntt=%233724&fromPage=plp&_requestid=333033
anti-FBXW7(ab109617/Abcam/Rabbit):https://www.abcam.com/fbxw7-antibody-ab109617.html
anti-GAPDH(#5174/CST/Rabbit):https://www.cellsignal.cn/products/primary-antibodies/gapdh-d16h11-xp-rabbit-mab/5174?site-search-type=Products&N=4294956287&Ntt=%235174&fromPage=plp&_requestid=333097
anti-TBL1XR1(ab190796/Abcam/Rabbit):https://www.abcam.com/tblr1tbl1xr1-antibody-epr16153-ab190796.html

The following antibodies have been approved by the manufacturers for IF assay:
anti-OTUD3(HPA028543/Sigma-Aldrich/rabbit):https://www.sigmaaldrich.cn/CN/zh/product/sigma/hpa028543?context=product
anti-ZFP36(ab124024/Abcam/mouse):https://www.abcam.com/tristetraprolinttp-antibody-oti8b5-ab124024.html
anti-PDPN(ab10288/Abcam/mouse):https://www.abcam.com/podoplanin--gp36-antibody-18h5-bsa-and-azide-free-ab10288.html
anti-LYVE1(#67538/CST/rabbit):https://www.cellsignal.cn/products/primary-antibodies/lyve-1-e3l3v-rabbit-mab/67538?site-search-type=Products&N=4294956287&Ntt=%2367538&fromPage=plp&_requestid=333628

The following antibodies have been approved by the manufacturers for PLA assay:
anti-OTUD3(HPA028544/Sigma-Aldrich/rabbit):https://www.sigmaaldrich.cn/CN/zh/product/sigma/hpa028544?context=product
anti-ZFP36(ab124024/Abcam/mouse):https://www.abcam.com/tristetraprolinttp-antibody-oti8b5-ab124024.html

The following antibodies have been approved by the manufacturers for IP assay: √
anti-OTUD3(MABS1819M/Sigma-Aldrich/mouse):https://www.sigmaaldrich.cn/CN/zh/product/mm/mabs1819m?context=product
anti-Flag(F7425/Sigma-Aldrich/rabbit):https://www.sigmaaldrich.cn/CN/zh/product/sigma/f7425?context=product
anti-ZFP36(ABE285/Merck Millipore/rabbit):https://www.sigmaaldrich.cn/CN/zh/product/mm/abe285?context=product
anti-MYC(#2276/CST/mouse):https://www.cellsignal.cn/products/primary-antibodies/myc-tag-9b11-mouse-mab/2276?site-search-type=Products&N=4294956287&Ntt=%232276&fromPage=plp

The following antibodies have been approved by the manufacturers for RIP assay: √
anti-ZFP36(ABE285/Merck Millipore/rabbit):https://www.sigmaaldrich.cn/CN/zh/product/mm/abe285?context=product
anti-CNOT1(A305-787A/Bethyl/Rabbit):https://www.thermofisher.cn/cn/zh/antibody/product/CNOT1-Antibody-Polyclonal/A305-787A-M?adobe_mc=MCMID%7C23008382237272968250991088604612048510%7CMCAID%3D2FF184BE0515F45C-6000092D8EA3C070%7CMCORGID%3D5B135A0C5370E6B40A490D44%40AdobeOrg%7CTS=1614293705
anti-IgG(ab172730/Abcam/rabbit):https://www.abcam.com/rabbit-igg-monoclonal-epr25a-isotype-control-ab172730.html

The following antibodies have been approved by the manufacturers for CHIP assay: √
anti-FOXO1(#2880/CST/rabbit):https://www.cellsignal.cn/products/primary-antibodies/foxo1-c29h4-rabbit-mab/2880?site-search-type=Products&N=4294956287&Ntt=%232880&fromPage=plp&_requestid=332406
anti-p300(ab275378/Abcam/rabbit):https://www.abcam.com/kat3b--p300-antibody-epr23495-268-chip-grade-ab275378.html
anti-H3K27ac(ab4729/Abcam/rabbit):https://www.abcam.com/histone-h3-acetyl-k27-antibody-chip-grade-ab4729.html
anti-Polymerase II(ab264350/Abcam/rabbit):https://www.abcam.com/rna-polymerase-ii-antibody-ab264350.html
anti-IgG(ab172730/Abcam/rabbit):https://www.abcam.com/rabbit-igg-monoclonal-epr25a-isotype-control-ab172730.html

Eukaryotic cell lines

Policy information about cell lines

Cell line source(s)

The human esophageal cancer cell lines, including KYSE180, KYSE520, KYSE140, KYSE30, KYSE410, KYSE510, were obtained from Deutsche Sammlung von Mikroorganismen und Zellkulturen (DSMZ, Braunschweig, Germany), the German Resource Centre for Biological Material.The human normal esophageal epithelial cells (NEECs) were purchased from ScienCell (Carlsbad, CA, USA).293T cells were obtained from the Cell Bank of Shanghai Institutes of Biological Sciences (Shanghai,

	China).
Authentication	Cell lines were authenticated by short tandem repeat (STR) fingerprinting.
Mycoplasma contamination	All cell lines tested negative for mycoplasma contamination.
Commonly misidentified lines (See ICLAC register)	No cell lines uesd in this study is listed in the database of commonly misidentified cell lines maintained by ICLAC.

Animals and other organisms

Policy information about studies involving animals; ARRIVE guidelines recommended for reporting animal research

Laboratory animals	BALB/c-nude mice (Male, 4-5 weeks old) were purchased and housed in barrier facilities on a 12 h light/dark cycle at temperature 18–22 °C and humidity 50–60%.
Wild animals	The study did not involve wild animals
Field-collected samples	The study did not involve field-collected samples.
Ethics oversight	All experimental procedures were approved by the Institutional Animal Care and Use Committee of Sun Yat-sen University and performed following the Declaration of Helsinki.

Note that full information on the approval of the study protocol must also be provided in the manuscript.

Human research participants

Policy information about studies involving human research participants

Population characteristics	This study used 228 paraffin-embedded esophageal cancer patient specimens that had been clinically and histopathologically diagnosed at the Sun Yat-sen University Cancer Center from 2008 to 2015.Details of the covariate-related population characteristics of human research participants (such as age, gender, smoking habbit, etc.) were provided in the Supplementary Table 1.
Recruitment	We randomly selected 228 patients with complete basic information. There is no potential self-selection bias or other biases.
Ethics oversight	Ethics approval was obtained from the Institutional Research Ethics Committee of Sun Yat-sen University Cancer Center with approved number #GZR2016-111.

Note that full information on the approval of the study protocol must also be provided in the manuscript.
